# Elucidation of microstructural changes in leaves during senescence using spectral domain optical coherence tomography

**DOI:** 10.1038/s41598-018-38165-3

**Published:** 2019-02-04

**Authors:** Tulsi Anna, Sandeep Chakraborty, Chia-Yi Cheng, Vishal Srivastava, Arthur Chiou, Wen-Chuan Kuo

**Affiliations:** 1 National Yang-Ming University, Biophotonics and Molecular Imaging Research Center, Taipei, 11221 R.O.C. Taiwan; 2 National Taiwan University, Graduate Institute of Photonics and Optoelectronics, Taipei, 10617 R.O.C. Taiwan; 3 Thapar University, Electrical and Instrumentation Engineering Department, Patiala, 147004 India; 4 National Yang-Ming University, Institute of Biophotonics, Taipei, 11221 R.O.C. Taiwan

## Abstract

Leaf senescence provides a unique window to explore the age-dependent programmed degradation at organ label in plants. Here, spectral domain optical coherence tomography (SD-OCT) has been used to study *in vivo* senescing leaf microstructural changes in the deciduous plant *Acer serrulatum Hayata*. *Hayata* leaves show autumn phenology and change color from green to yellow and finally red. SD-OCT image analysis shows distinctive features among different layers of the leaves; merging of upper epidermis and palisade layers form thicker layers in red leaves compared to green leaves. Moreover, A-scan analysis showed a significant (p < 0.001) decrease in the attenuation coefficient (for wavelength range: 1100–1550 nm) from green to red leaves. In addition, the B-scan analysis also showed significant changes in 14 texture parameters extracted from second-order spatial gray level dependence matrix (SGLDM). Among these parameters, a set of three features (energy, skewness, and sum variance), capable of quantitatively distinguishing difference in the microstructures of three different colored leaves, has been identified. Furthermore, classification based on k-nearest neighbors algorithm (k-NN) was found to yield 98% sensitivity, 99% specificity, and 95.5% accuracy. Following the proposed technique, a portable noninvasive tool for quality control in crop management can be anticipated.

## Introduction

The lifespan and microstructure of leaves are crucial for the overall development of plants. Leaf growth involves both maintenance and senescence^1^. The self-maintenance activity gradually decreases during the life span, while the senescence activity proceeds till the abscission or death of the leaves^2^. Senescence in plants, corresponds to both degenerative and recycling process (*viz*. nitrogen is recycled to stems from leaves for their growth) and is a programmable development in leaves^1,3,4^. Leaf senescence, which is an organ level senescence in a plant, is manifested, in general, by the changes in its color from green, to yellow. The initiation and progression of senescence are governed by several environmental factors such as temperature, humidity, nutrition, shading, and oxidative stress, etc.^5^. For example, during autumn phenology in deciduous plants, the initiation of senescence shows a spectacular change of colors from green, to yellow, orange and finally red^6^. Apart from these, internal factors such as reproductive development, hormonal changes, phytochrome levels, etc. also influence the senescence activity^7^. Further, loss of photosynthetic activity might also initiate senescence in leaves. Leaf senescence has both good and adverse effects on plants such as the crops yield and postharvest loss of vegetables^2^. Hence, understanding of the key factors associated with senescence will provide valuable information for agricultural sciences. In addition, plant leaves are easily accessible for experimental assays with reproducible life history and provide a very unique window for detail study of the senescence.

Till date, several traditional biochemical techniques have been used to study the changes in various chemical biomarkers for leaf senescence such as chlorophyll content, proteins, lipids, and other associated acids^8–11^. However, these techniques depend on cell lysis and inapplicable for *in vivo* studies. In the alternative, microstructural biomarkers have also been identified to study the leaf development stage^8–11^. Average leaf mass per unit area and parenchyma thickness are commonly used as microstructural biomarkers. Changes in leaf microstructure are closely associated with the photosynthetic activity, which usually degrades during senescence^12^. Imaging modalities such as scanning electron microscopy (SEM)^13^, transmission electron microscopy (TEM)^14^ and X-ray computed tomography^15^ have been widely used for quantitative visualization microstructural biomarkers in leaves. However, these specialized techniques need detail sample preparations and also not suitable for *in vivo* applications. Complementary optical imaging platforms such as confocal and multiphoton fluorescence microscopy^16^, hyperspectral reflectance microscopy^17^, Fourier transform infrared spectroscopy (FTIR)^18^, and Raman spectroscopy^19^ etc. are valuable for non-destructive study of leaf senescence at the cellular, molecular, as well as microstructural level. However, though these techniques provide interesting findings, are limited by penetration depth and sometimes needs physical sectioning. Therefore, non-destructive and fast optical imaging modality with high penetration depth to monitor the microstructural changes in leaves during senescence is required.

Optical coherence tomography (OCT), based on low coherence interferometry, is a label-free optical technique which can provide high-speed three dimensional (3D) cross-sectional imaging with micrometer resolution at depth (on the order of several millimeters) in highly scattering samples^20^. In addition, OCT also possesses several advantages over other optical imaging modalities such as: miniaturization with fiber-based optics, compatibility with functional imaging and data reconstruction algorithm, use of infrared light sources to increase penetration depth by reducing light scattering and absorption in tissues^21^. Till date, OCT has found applications in numerous biomedical studies, *viz*. ophthalmic imaging, retinal surgery, dermatology, forensic sciences, etc. and non-destructive testing (NDT) since its first realization in 1991^20–22^.

Apart from its potential biomedical applications, OCT has found little use in understanding problems in plant biology and agriculture. OCT has been reported to elucidate qualitatively the microstructural changes in the layers of fruits, seeds and other parts of plants^23,24^. OCT has also been applied to detect cucumber green mottle mosaic virus (CGMMV) in cucumber seeds, and to study microstructural changes in infected apple leaves^25,26^. Recently, spectral domain OCT (SD-OCT) has been used for *in vivo* monitoring of growth of *Capsicum annuum leaf* and fungal infection in leaves^27^. A compact SD-OCT with a spectrometer for *in situ* leaf quality assessment has also been reported^28^. Thus, SD-OCT and OCT, in general, have the potential to become an invaluable tool in agriculture and plant science.

In this study, SD-OCT has been used to obtain 3D cross-sectional images of *Acer serrulatum Hayata* leaves to illustrate and quantify the leaf microstructural changes during senescence (due to seasonal change; autumn phenology). Moreover, total chlorophyll content of the leaves at different stages of the developmental senescence has been studied. *Acer serrulatum Hayata* is a broadleaf deciduous tree which belongs to the species maple, and it shows remarkable autumn phenology. Here, we compared the microstructural changes in leaves among different stages of leaf development and senescence, i.e., green, yellow, and red leaves. 3D cross-sectional images and the corresponding attenuation coefficient (of light) showed remarkable microstructural changes among these different colored leaves. Further, to quantify these changes, we have applied textural analysis algorithms (Spatial-gray level dependent matrix; SGLDM) to extract fourteen quantitative parameters to distinguish senescing leaves from non-senescing^29,30^. Our results further show lower total chlorophyll content in the senescing leaves. To the best of our knowledge, this is the first reported study of combining SD-OCT imaging with textural analysis algorithm in elucidating parametric quantification of leaf microstructure in senescence, a phenomenon of utmost importance from an agricultural perspective.

## Results

The key results of our SD-OCT-based study of the influence of senescence in the structured layers of leaves of deciduous plant ‘*Acer serrulatum Hayata*’, are presented below in this section.

### 2D and 3D image analysis of senescing leaves

**Figure 1:**
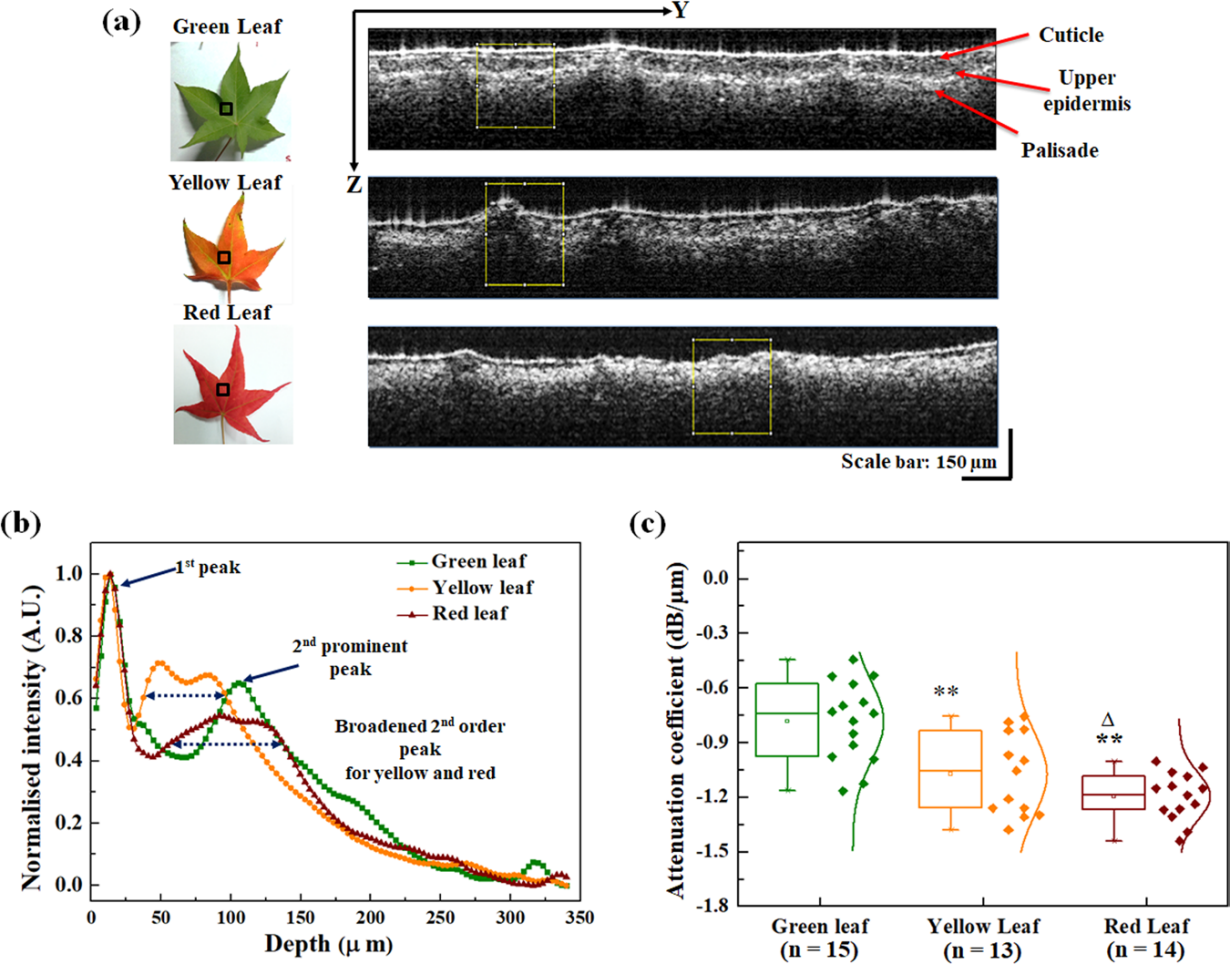
Two-dimensional (2D) SD-OCT image analysis. (**a**) Photographs of representative green, yellow and red leaves and the corresponding 2D SD-OCT cross-sectional images. Here, the black boxes in the leaf photographs are the approximate areas where the SD-OCT imaging is performed; while yellow boxes in the cross-sectional images are considered for the A-scan analysis and attenuation coefficients calculations. (**b**) Normalized A-scan analysis of leaves. (**c**) Box plot of the attenuation coefficients of light in green, yellow and red leaves. Median is shown as the horizontal line within the box; boundaries of the box indicate the 25th- and 75th -percentile, while the line extended from the box on both sides represent the extent of area where the outliers can be found. Data points with a normal distribution curve are also shown for each box. Student’s unpaired two-tailed t-test statistical significance: “**”p < 0.001 for green leaves vs. other conditions; “Δ”p < 0.05 for yellow vs. red leaves; “n” represents the number of data points.

In this work, three different colored *Hayata* leaves *viz*. green, yellow and red were chosen as three distinct stages of developmental senescence. To confirm the consistency during our experiments for the three categories, all the leaves were imaged on adaxial surface. Figure 1(a) shows the representative 2D SD-OCT cross-sectional depth images along with the photographs of the green, yellow and red leaves. The photographs emphasize the topographical changes among all the three categories. In addition, the 2D cross-sectional images clearly show the green leaf with three distinct layers *viz*. cuticle, upper epidermis, and palisade layers, however, for yellow and red leaves distinct demarcation of upper epidermis and palisade layers become indistinguishable (Fig. 1(a)), resulting in a single thickened layer. Thus, this result strongly reflects the alteration of structured layers of leaves during the development of senescence and suggests that SD-OCT characterization can be potentially applied in this kind of study.

Leaves are multilayered structures with variation in absorption coefficients in different layers; nevertheless, “A-scan” and “B-scan” analysis was performed to obtain more detail microstructural information from the SD-OCT images. In OCT, A-scan is referred for depth scan (which is related to longitudinal scan); while B-scan is referred to sagittal, or transverse sections such as XZ, or YZ plane. The thickness between two layers of a leaf can be defined through the corresponding distance between the A-scan profile (amplitude scan) peaks. Normalized “A-scan analysis” of leaves at different senescence stages and corresponding A-scan intensity plot are shown in Fig. 1(b). Equal areas (~250 µm in the lateral direction) were used for A-scan analysis of all the leaves samples. A convolution filter was used to compensate for speckle noise in A-scan signals to avoid erroneous peak detection. Hundred successive A-scan signals were extracted from a 2D cross-sectional image (B-scan images), averaged and normalized by the maximum value. The maximum value intensity peak in the axial direction of the image was identified as the first peak. The peaks in the A-scan plot of the green leaves shows different layers of the leaves; the broadening of the second order peak in yellow and red leaves substantiates the observation in Fig. 1(a) that the upper epidermis and palisade layers merged to form a thick layer in these leaves. Hence, the increase in thickness and merging of epidermis and palisade layers can be considered as one of the initial symptoms in the senescence progression. So, it might be useful to identify the leaf layer structure changes to understnad the physical status of the leaves during senescence.

To obtain far more valuable structural information and quantify the changes above in terms of intensity, attenuation coefficient was also calculated for each case (Fig. 1(c)). The attenuation coefficient is an intrinsic optical property of tissues that is strongly related to the OCT signals. It can be defined as the amount of light scatter and absorption per unit distance as light travels in a tissue or semitransparent material. For the calculation of the attenuation coefficient, the chosen area of interest was taken 10 pixels below the air-sample surface interface to avoid high-intensity reflections and fluctuations. For image analysis, 200 B-scan images were taken to obtain an averaged B-scan image; subsequently, 100 adjacent A-lines (corresponding to a lateral scan length of ~492 µm) were chosen from the center of this averaged B-scan image and averaged. The depth-dependent reflectivity or A-line scan was obtained from this averaged A-line; corresponding to an axial region of ~320 µm. A linear-fitting model was used to obtain the slope of the attenuation of each A-line. The calculated mean attenuation coefficients ± SEM (standard error of mean) for green, yellow and red leaves are: −0.78 ± 0.05 dB/µm (n = 12), −1.10 ± 0.06 dB/µm (n = 11), and −1.20 ± 0.03 dB/µm (n = 10), respectively (Fig. 1(c)). Our result thus shows a statistically significant lower attenuation coefficient for the yellow and red leaves as compared to green leaves. The higher value of slope corresponds to a higher attenuation of light for the same penetration depth.

**Figure 2:**
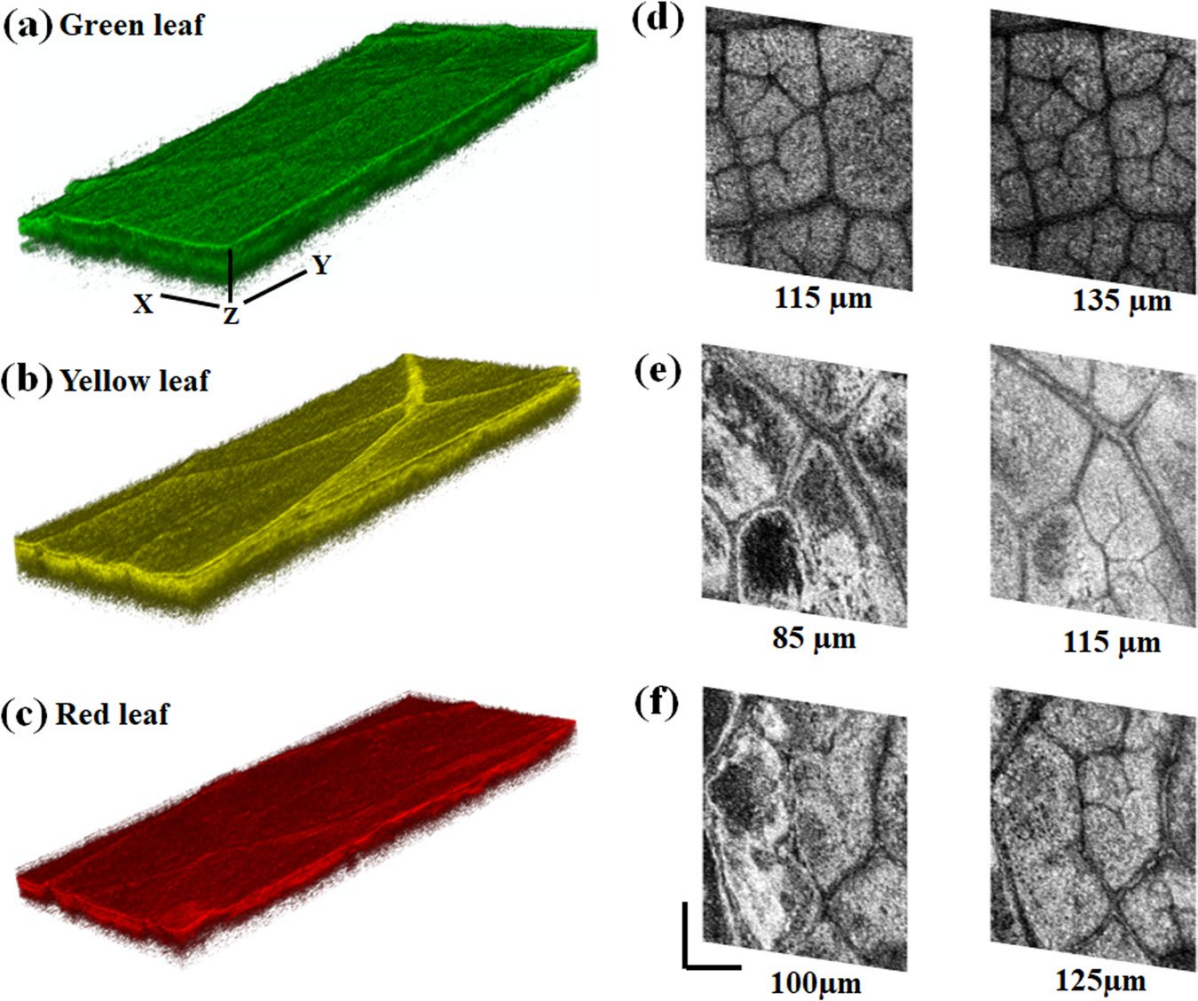
Three-dimensional (3D) and *en face* SD-OCT images of the green, yellow and red colored leaves. (**a**–**c**) Show the pseudocolored 3D (2 mm × 2 mm × 350 µm) reconstructed images of the green, yellow and red colored *Hayata* leaves. *En face* images (shown in gray color) are at (**d**) 115 and 135 µm depths in green leaf; (**e**) 85 and 115 µm depths in yellow leaf; and (**f**) 100 and 125 µm depths in red leaf. Scale bar: 500 µm.

Figure 2 shows the 3D, and *en face* (XY) oriented images of leaves at different conditions. Figures 2(a–c) show the 3D reconstructed images of the green, yellow and red colored leaves respectively. The selected 3D reconstructed volume was 2 mm × 2 mm × 350 µm. From these images, distinguishable microstructural differences are not observed at the upper cuticle layer among the three conditions of leaves. This substantially validates the existence of sharp first peak as can be seen in the A-scan analysis plot (Fig. 1(b)). Further, *en face* sectional images of the green leaf at depths 115 and 135 µm, yellow leaf at 85 and 115 µm, and red leaf at 100 and 125 µm are shown in Figs. 2(d–f), respectively. These images, at different depths between the upper epidermis and palisade layer, corresponds to the second peak in the A-scan analysis plot in Fig. 1(b). The *en face* images clearly show the microstructural changes in the upper epidermis and palisade layer among green, yellow and red leaves. Our results thus further validate the fact that microstructural changes in upper epidermis (protects leaves from pathogens as well as other environmental factors), as well as stoma also contribute to the progression of senescence. To further quantify the changes in the microstructural features due to senescence in *Hayata* leaves, we adopted a texture analysis procedure; the results are presented below.

### Texture parameter quantifications

In this study, fourteen texture parameters were evaluated for green, yellow and red colored *Hayata* leaves. A total of 44 green, 43 yellow, and 56 red leaves were imaged *in vivo* using the SD-OCT system. In a B-scan image of 813 × 750 pixels (width × height), an ROI of 200 × 200 pixels sub-image was chosen to correspond to a physical area of 492 µm × 533 µm. These sub-images were then processed using SGLDM, and each feature was extracted.

**Figure 3:**
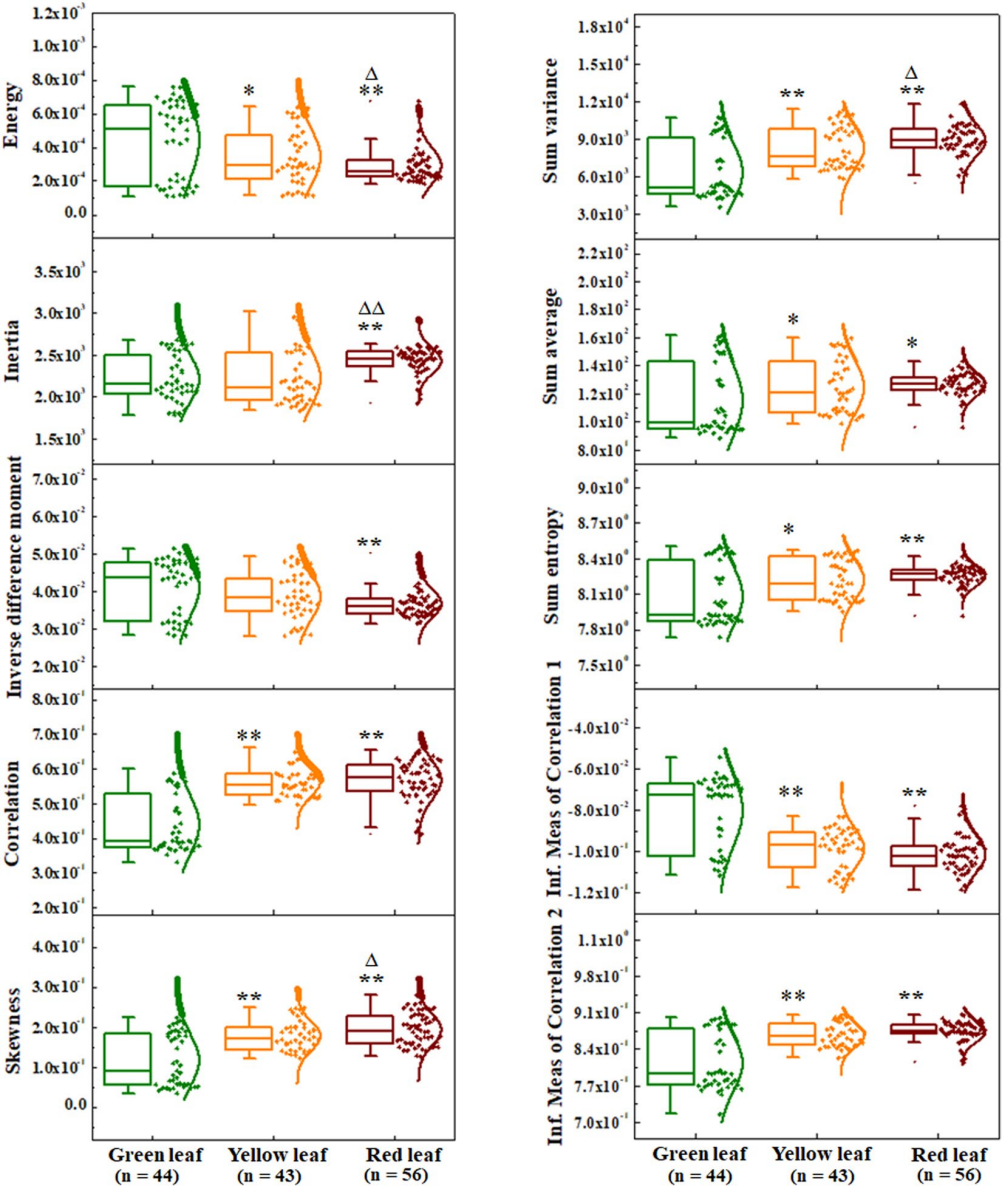
Box plots for the comparison of texture parameters using spatial gray-level dependence matrix (SGLDM). The horizontal line within the box indicates the median, boundaries of the box indicate the 25^th^- and 75^th^ -percentile, and the line extended form the box in both sides represent the extent of the area where the outliers can be found. Data point distribution with a solid curve (to show normal data distribution) is also shown for each box. Textural parameters, *viz*. energy, inertia, inverse difference moment, correlation, skewness, sum variance, sum average, sum entropy, and information measure of correlation 1 and 2 among the green, yellow and red leaves, extracted from the SD-OCT images of the three different colored leaves are shown. Y-axis for each parameter are shown in arbitrary units (A.U.) as it was obtained from pixel gray level values. Student’s unpaired two-tailed t-test statistical significance: “*”p < 0.05; “**”p < 0.001 for green leaves vs. other conditions; “Δ”p < 0.05; “ΔΔ”p < 0.001 for yellow vs. red leaves; “n” represents the number of data points.

Figure 3 summarizes the changes of most significant ten texture parameters; while Supplementary Fig. S1 shows the other four parameters as a reference. These texture parameters help to understand the microstructural changes among the green, yellow and red leaves. The mathematical expression and their significance are succinctly summarized in Supplementary Table S1. From Fig. 3, it is easily discernable that apart from the texture parameters inertia and inverse difference moment which can only distinguish between green and red leaves, the other parameters significantly distinguish the green leaves from yellow and red leaves. However, inertia can also differentiate (with p < 0.001) yellow and red leaves. Most interestingly, the texture parameters energy, skewness and sum variance can successfully quantify the microstructural differences among all the leaves with different colors. The parameter energy decreased to ~22% (with p < 0.05) and ~34% (with p < 0.001) for yellow and red leaves vs. green leaves respectively; while the energy for red leaves decreased by ~14% (with p < 0.05) against the yellow leaves. Skewness increased by ~50% (with p < 0.001) and ~63% (with p < 0.001) for yellow and red leaves as compared with green leaves respectively (Fig. 3); also a ~10% (with p < 0.05) increment of skewness was also observed for red leaves in comparison with yellow leaves. Further, the texture parameter sum variance showed an increase of ~30% (with p < 0.001) and ~40% (with p < 0.001) for yellow and red leaves in comparison with green leaves while it showed a ~10% (with p < 0.05) change between yellow and red leaves. Hence, quantitative evaluation of textural features can help us to distinguish the young and healthy leaves from the senescing leaves.

**Figure 4:**
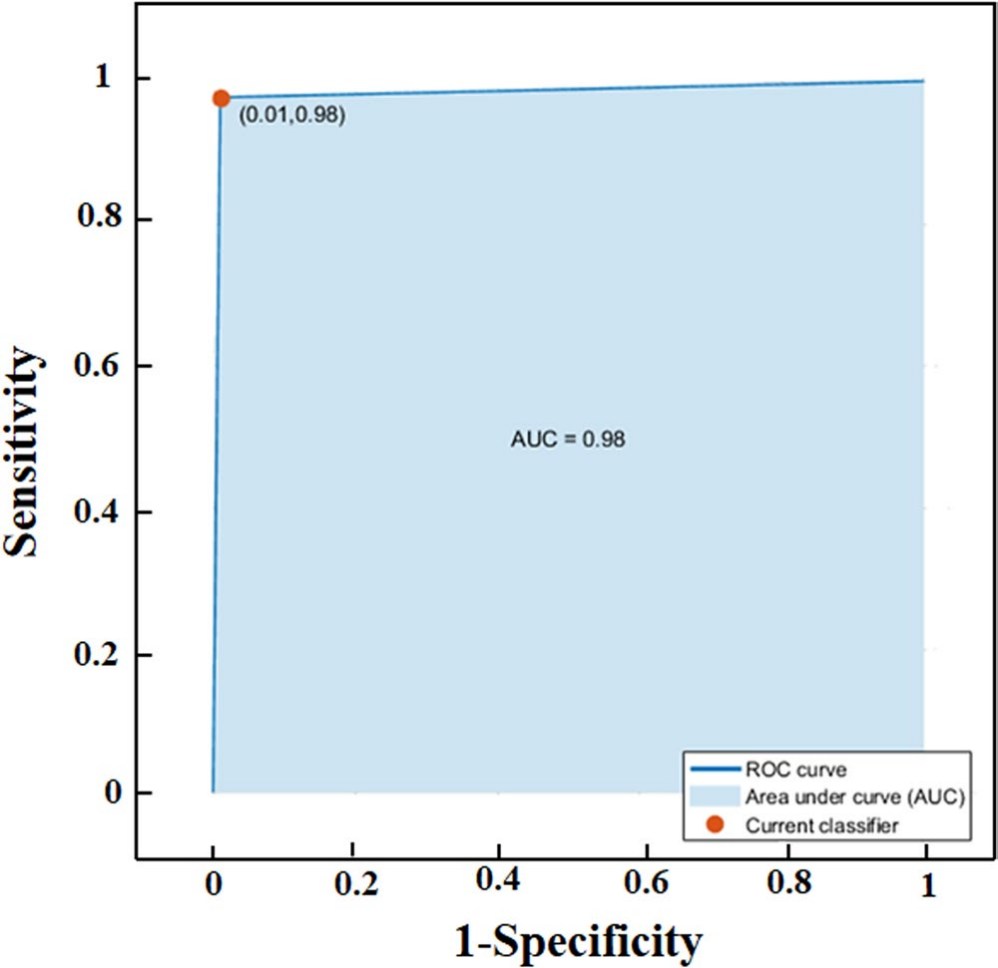
The receiver operating characteristics (ROC) curve to show the accuracy of the k-NN classifier. ROC curve for the significant texture features (energy, inverse difference moment, skewness, and sum variance) for the classification of green, yellow and red leaves are shown. The area under the curve (AUC) is 0.98.

Furthermore, to validate the capability of SGLDM model to differentiate between green, yellow and red leaves, the widely used classifier k-nearest neighbors algorithm (k-NN), a non-parametric technique, was chosen. In k-NN classification, the input consists of the k closest training examples in the feature space; while the output comprises the class membership. An object is classified by a majority vote of its neighbors, with the object assigned to the class most prevalent amongst its k-nearest neighbors. The accuracy of the classifier is determined by the area under the receiver operating characteristics (ROC). Figure 4 shows the ROC for the textural features (energy, inverse difference moment, skewness, and sum variance) to distinguish the three different colored leaves simultaneously. The calculated sensitivity, specificity, and accuracy of the applied textural analysis procedure were ~98%, ~99%, and ~95.5%, respectively, for the selected features. These values suggest that SD-OCT imaging, in conjunction with SGLDM textural analysis, have a strong potential to quantitatively classify different stages of senescence in leaves.

**Figure 5:**
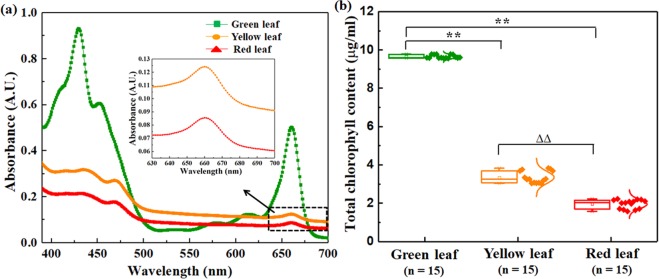
Total chlorophyll content determination. (**a**) Absorbance curves of the extracts of green, yellow, and red *Hyata* leaves in 99% acetone. The plot in the inbox shows the enlarged view of the absorption peaks (identified as the region of yellow and red leaves in the 630–700 nm wavelength range). (**b**) Box plot of the total chlorophyll contents in the three different colored leaves. Horizontal line within the box indicates the median, the boundaries of the box shows the 25^th^- and 75^th^- percentile; while the extended line from the sides of the box indicate the extent of area where the outliers can be found. Student’s unpaired two-tailed t-test statistical significance: “**”p < 0.001 for green leaves vs. other conditions; “ΔΔ”p < 0.001 for yellow vs. red leaves; “n” represents the number of data points.

As discussed previously, morphological changes are also accompanied by the changes in the chlorophyll content of the senescing leaves. Thus, to validate our findings using SD-OCT with chlorophyll contents in the leaves, the absorbance curves of the total pigment extracts of the three different colored leaves in acetone were recorded (Fig. 5(a)). Subsequently, following Bruinsma’s protocol, the total chlorophyll concentration of the leaves were determined^31,32^. The detail of the sample preparation can be found in the “*Materials and methods*” section. Our results showed that the total chlorophyll concentrations (in µg/ml) of the yellow and red leaves are significantly decreased (p < 0.001) compared to the green leaves by ~65.29% and ~79.80% respectively; further, the chlorophyll content decreases by ~41.77% (p < 0.001) for red leaves compared to yellow leaves (Fig. 5(b)). These findings further confirm that the total chlorophyll content of senescing leaves at different developmental stages varies significantly and can be correlated with the changes in the microstructural changes in the leaves.

## Discussions

We have investigated the changes in microstructural features associated with the senescence (due to autumn phenology) of leaves using SD-OCT. The A- and B- scan (Fig. 1) and 3D SD-OCT (Fig. 2) images for different conditions of leaves provide the optical visualization of qualitative changes in microstructural features; further determination of the attenuation coefficient of light (Fig. 1(c)) and utilization of texture analysis algorithm (Fig. 3) help to quantify those changes. In addition, the chlorophyll content of the different stages of the leaves (Fig. 5) was also evaluated.

Senescence is considered as the final stage of leaf development and strongly reflects the process towards death. Thus, studying the structural features changes during this process will have a profound impact in agricultural and allied sciences. Senescence is associated with the down-regulation of photosynthesis apparatus and corresponds to an alteration of layered structural changes in leaves^1,10,12^. Our study using SD-OCT also shows prominent changes in the layered organization and total chlorophyll content of the *Hayata* leaves. For the red and yellow leaves, the upper epidermis and palisade layers merged and a thick layer is observed which is distinctly absent in green leaves (Fig. 1(b)). Similar findings also can be found in leaves affected by pathogen attacks, which leads to senescence^9,33^. Thus, it can be hypothesized that senescence due to pathogen attacks and autumn phenology might have similar structural alterations. Calculation of the attenuation coefficient from A-scan images also shows the distinct structural changes among green *vs*. yellow and red leaves (Fig. 1(c)). Higher attenuation coefficient for green leaves as compared with the other leaves shows the intact layered structure corresponding to their healthy status. This is the first reported validation of layered structural changes in senescing leaves due to the seasonal variation in autumn.

To further substantiate these observations, textural parameters for the green and senescing leaves were evaluated. Our results show that energy, skewness and sum variance can significantly distinguish the three kinds of *Hayata* leave samples (green, yellow, and red) chosen in this study. Energy defines the intensity homogeneity in an image, reflecting pixel-pair repetitions in an image plane. Thus the lower value of energy reflects structural asymmetry. On the other hand, skewness defines the imbalance between the gray levels among pixels and their mean values. Thus, higher skewness also corresponds to the asymmetric structure. Sum variance, which is a measure of heterogeneity, defines the sum distribution of pixel gray level values around their mean. This feature was also reflected by the texture parameter inverse difference moment. Our results show that energy decreases while skewness and sum variance increases from green to red leaves (Fig. 3), thus corroborating the conclusion that in leaf senescence, the leaf microstructure becomes more asymmetrical and/or heterogeneous compared with those in young and healthy conditions. Furthermore, inverse difference moment change also reflects a similar conclusion for green and red leaves (Fig. 3).

In addition to microstructural studies with SD-OCT, total chlorophyll content, an important physiological parameter associated with senescence, changes statistically significantly with the advanced stages of senescence (Fig. 5). Existing literatures also show the change in chlorophyll content and its correlation with leaf structure (leaf thickness, leaf mass area, and leaf mass density) alterations^34^. However, these parameters, which are generally considered for leaf structure, are macroscopic. On the other hand, our approach of SD-OCT can provide information of leaf microstructure at submicron resolution non-invasively. In addition, our finding also corroborates well with existing literature where it has also been shown that lower chlorophyll content and leaf structure changes associates well with senescence^12,34^. Thus, we can view our findings in the light that SD-OCT structural analysis along with total chlorophyll content might serve as complementary to each other in the understanding of the progression of senescence.

Senescence is a progressive developmental process which terminates with the death of the organ (*viz*. leaf), or the plant as a whole. It starts with the early stages of development, and the intermediate stage of the process is highly undetermined^4^. In this study, we have chosen three distinct major time points (as reflected by green, yellow and red colored leaves) of leaf senescence in developmental context to correlate our experimental results with the progression of the senescence in *Hayata* leaves. Here, the yellow color leaves are chosen as a representative intermediate stage between the healthy green leaves and the senescing red colored leaves, a stage showing the peak of the senescence. This undetermined nature of senescence stages apart from terminate one, can also be observed in the results of textural features for the three different colored leaves (Figs. 3 and S1). For the green and red leaves, all the parameters can significantly distinguish these two end-point stages (Fig. 3). However, the ROC curve analysis showed that the features skewness, energy, and sum variance could successfully categorize the selected different stages of senescence in leaves simultaneously with high accuracy, specificity and sensitivity (Fig. 4). Our approach is thus capable of quantitatively discriminating senescing leaves from healthy leaves due to autumn phenology.

## Conclusions

SD-OCT is a highly robust, non-destructive and informative technique that has, till date, limited application in plant science. Moreover, this study is the first of its kind in applying textural analysis algorithm to analyze SD-OCT images to quantify the microstructural changes involved in the senescence of leaves. SD-OCT techniques, equipped with near-infrared (NIR) light source, provide higher penetration in highly scattering samples such as leaves with little or no phototoxicity. *Acer serrulatum Hayata* plants have been widely planted in Taiwan as a part of the pollution control measure in cities which provides a strong motivation for this study. Our approach can complement traditional approaches to study this kind of problems. In field studies, such as in agriculture, a leaf can be easily sacrificed without affecting the yield of crops, and our approach can be utilized to predict the health of the crops. Furthermore, senescence and death in leaves are active development strategies that contribute to the survival of the plant. The work presented here thus can, in future, be implemented to tackle basic and practical problems in plant physiology and pathology, such as senescence and death in leaves caused by different agents (pathogen attack, pollution etc.).

## Materials and Methods

### Leaf sample preparation

In this study, leaves were collected fresh (on the day of experiment) from an *Acer serrulatum Hayata* tree grown in the campus of National Yang-Ming University, Taipei, Taiwan during the months from September to late November. Leaves were plucked from three selected trees to maintain the similar conditions at a particular time of the day (sunny days preferred). We chose three different colorations (green, yellow and red) of leaves signifying different stages of senescence. Care was taken to avoid any leaves infected by pathogens. A small section of the leaf was cut and mounted on a glass slide for SD-OCT imaging.

To obtain the chlorophyll absorbance curves of the leaves, small pieces (1 cm × 1 cm) leaf tissues were cut and homogenized with 1 ml of 99% acetone in a pestle. To remove the remaining debris, the homogenate is filtered using centrifugation at 1000 g for 5 mins. The chlorophyll absorbance curves were immediately determined using DU 800 spectrophotometer (Beckman coulter, Fullerton, Germany).

### Experimental set up of SD-OCT

**Figure 6:**
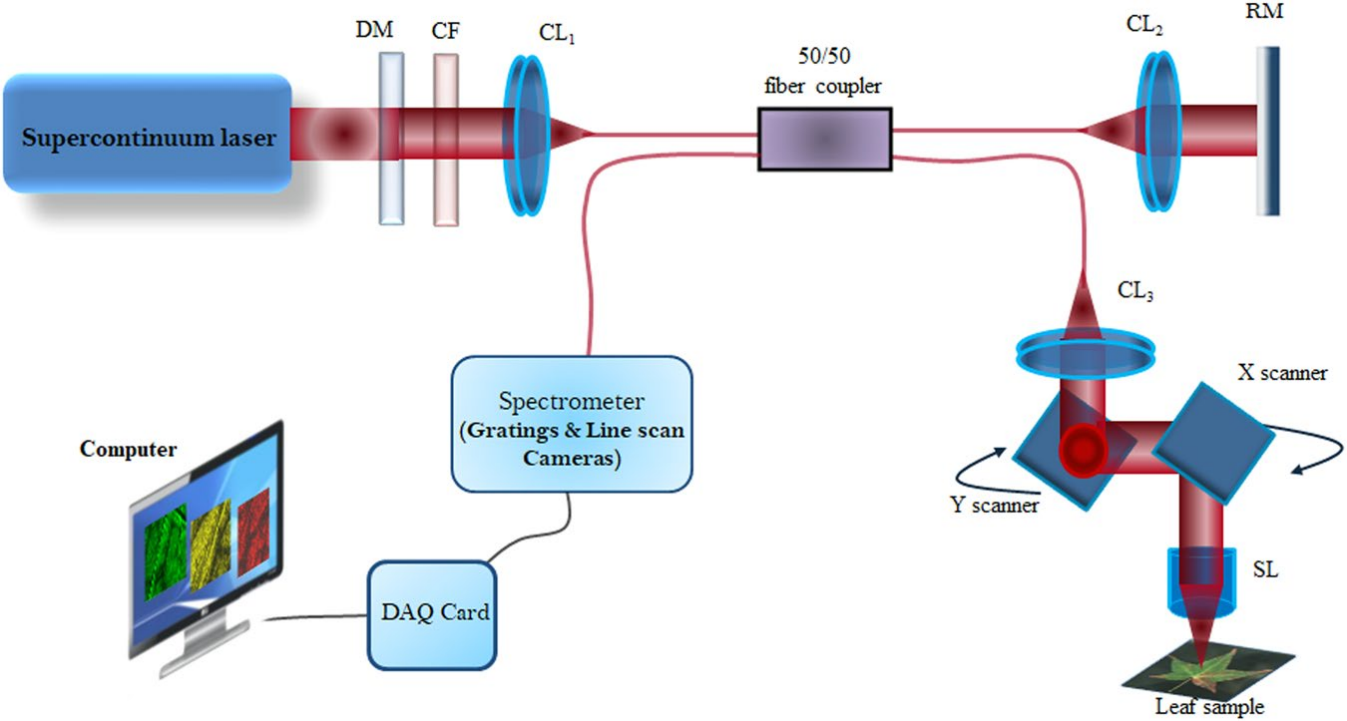
A schematic of SD-OCT set up. DM: Dichroic mirror; CF: Color filter; CL: Collimating Lens; RM: Reference mirror; SL: Scan lens; DAQ card: Data acquisition card.

The schematic of the experimental set up of the SD-OCT system is shown in Fig. 6. The detail of the system can be found elsewhere^35^. In short, a broadband (wavelength range: 650–1800 nm) supercontinuum fiber laser (SuperK Extreme, NKT Photonics, Denmark) was used as an illumination source in the present system. Using an appropriate dichroic mirror (DM) and color filter (CF) set, the desired wavelength band of 1100–1550 nm (center wavelength: 1275 nm, full-width half maximum (FWHM): 240 nm) was chosen for our experimental measurements. This desired light was then incident into a fiber-optic based low coherence Michelson interferometer. A 50/50 fiber coupler was used to direct the light to the sample and reference arms of the interferometer. 2D galvo scanners (61710PS1XY2-S4, Cambridge Technology, U.S.A.) with scan lens (LSM03, EFL = 36 mm, NA = 0.05547) were used to scan the leaves samples. A 2 mm × 2 mm scanning range was used. The interference signal was recorded using a spectrometer (COBRA SWIR C-1155-1395-GL2, Wasatch Photonics, USA) with a 2048 pixels line scan CMOS sensor (GL2048L-10A-ENC-STD-210, Sensors Unlimited Inc., U.S.A.). With the present system configuration, images with 1024 × 1000 pixels could be achieved with maximum A-line scan rate of 76 Ks^−1^. However, for our experimental measurements, the acquisition time of each 2D image (B-scan) was 37.25 ms. Total 813 A-scans were used to produce one 2D image, and 1800 images were obtained to make one 3D image. Correspondingly; acquisition time of one 3D image was 67 s. The axial and lateral resolutions of the system at the sample surface were approximately 7 and 9 µm respectively. System control and signal acquisition were achieved using LabView software.

### Texture analysis of B-scan SD-OCT images

Texture analysis, comprising of a set of mathematical relations to quantify the variation of gray level in an image, was applied to characterize different micro-features in the intensity variations in the acquired 2D-images. Textures represent the spatial distribution of intensity in terms of pixels’ gray levels in a region; and hence a change in the spatial distribution of intensity directly reflects the change in texture of a sample. Several statistical approaches have been proposed till date to define textural parameters^29,30,36^. In this work, Gray-level co-occurrence matrix (GLCM), also known as spatial gray-level dependence matrix (SGLDM), was used to obtain Haralick’s textural features^30^. In SGLDM, co-occurrence matrices are constructed to obtain the changes in gray level in the region of interest (ROI). Here, the ROI was selected manually, and MATLAB-based analysis (MATLAB 2015b) was applied to obtain the 14 selected textural features^37^, summarized and defined in Supplementary Table S1.

### Statistical analysis

Student’s unpaired two-tailed t-test was performed to evaluate the significance of difference among different data points at different stages of leaves senescence. Null hypothesis was rejected for ‘p’ values lower than 0.05.

## Supplementary information

Supplementary Information
