# Supplementary material for: Elucidation of microstructural changes in leaves during senescence using spectral domain optical coherence tomography
Source: Sci Rep. 2019 Feb 4;9:1167. doi: 10.1038/s41598-018-38165-3 (PMC6362184; doi:10.1038/s41598-018-38165-3)
Supplement: Supplementary file 1 — Supplementary Information [file 41598_2018_38165_MOESM1_ESM.docx]

**Supplementary Information**

FULL ARTICLE

**Elucidation of microstructural changes in leaves during senescence using spectral domain optical coherence tomography**

Tulsi Anna^1†*^, Sandeep Chakraborty^1,2†^, Chia-Yi Cheng^1^, Vishal Srivastava^3^, Arthur Chiou^1,4^,

Wen-Chuan Kuo^1,4**^

^1^National Yang-Ming University, Biophotonics and Molecular Imaging Research Center, Taipei-11221, Taiwan, (R.O.C.).

^2^National Taiwan University, Graduate Institute of Photonics and Optoelectronics, Taipei-10617, Taiwan, (R.O.C.).

^3^Thapar University, Electrical and Instrumentation Engineering Department, Patiala-147004, India.

^4^National Yang-Ming University, Institute of Biophotonics, Taipei-11221, Taiwan, (R.O.C.).

^†^These authors contributed equally

*Corresponding author: e-mail: [tulsianna@gmail.com](mailto:tulsianna@gmail.com), Tel: +886-919572584.

**Corresponding author: e-mail: [wckuo@ym.edu.tw](mailto:wckuo@ym.edu.tw), Tel: +886-2-2826-7950.

**Figure S1.** **Box plots for the comparison of 4 selected texture parameters using spatial gray-level dependence matrix (SGLDM).** Texture parameters *viz.* entropy, difference variance, difference average, and difference entropy extracted from SD-OCT images of the green, yellow, and red leaves samples, are compared. The horizontal line within the box indicates the median, boundaries of the box indicate the 25^th^- and 75^th^ -percentile, and the line extended form the box in both sides represent the extent of the area where the outliers can be found . Data point distribution with a solid curve (to show normal data distribution) is also shown for each box. Student’s unpaired two-tailed t-test statistical significance: “*”: p < 0.05; for green leaves vs. other conditions; “Δ”: p < 0.05 for yellow vs. red leaves; “n” represents the number of data points.

**Table ST1. SDLGM based texture parameters, their respective mathematical formulae and description**

| Texture Parameters | Mathematical Expression | Description |
| --- | --- | --- |
| Energy | $\sum_{i=1}^{N} \sum_{j=1}^{N} p(i,j)^{2}$ | A measure of the uniformity (or orderliness) of the gray level distribution of the image |
| Entropy | $-\sum_{i=1}^{N} \sum_{j=1}^{N} p(i,j) \log[p(i,j)]$ | A measure of the degree of disorder among pixels in the image |
| Inertia | $\sum_{i=1}^{N} \sum_{j=1}^{N} (i-j)^{2}p(i,j)$ | A measure of the local variations of gray levels present in an image. |
| Inverse difference moment homogeneity), | $\sum_{i=1}^{N} \sum_{j=1}^{N} \left(p(i,j)/1+(i-j)^{2}\right)$ | A measure of the smoothness (homogeneity) of the gray level distribution of the image |
| Correlation | $\sum_{i=1}^{N} \sum_{j=1}^{N} \{(i-\mu_{x})(j-\mu_{y})/\sigma_{x}\sigma_{y}\}p(i,j)$ | A measure of the linear dependency of gray levels on those of neighboring pixels |
| Sum average | $\sum_{i=2}^{2N} ip_{x+y}(i)$ | A measure of the mean of the gray level sum  distribution of the image |
| Sum Entropy | $-\sum_{i=2}^{2N} p_{x+y}(i) \log\{p_{x+y}(i)\}$ | A measure of the disorder related to the gray level sum distribution of the image |
| Sum variance | $\sum_{i=2}^{2N} \left(i-\left[\sum_{i=2}^{2N} ip_{x+y}(i)\right]\right)^{2}$ | A measure of the dispersion (relative to the mean) of the gray level sum distribution of the image |
| Difference average | $\sum_{i=2}^{2N} ip_{x-y}(i)$ | A measure of the mean of the gray level difference distribution of the image |
| Difference variance | $\sum_{i=2}^{2N} \left(i-\left[\sum_{i=2}^{2N} ip_{x-y}(i)\right]\right)^{2}$ | A measure of the dispersion (relative to the mean) of the gray level difference distribution of the  Image |
| Difference entropy | $-\sum_{i=0}^{N-1} p_{x-y}(i) \log\{p_{x-y}(i)\}$ | A measure of the disorder related to the gray level  difference distribution of the image |
| Information measure of correlation 1 | $HXY-HXY1/\max\{HX-HY\}$ | A measure of Correlation involving entropies |
| Information measure of correlation 2 | $\left(1-\exp[-2(HXY2-HXY)]\right)^{1/2}$ | A measure of Correlation involving entropies |
| Skewness | $\sum_{i=1}^{N} \sum_{j=1}^{N} \left[\{(i-\mu_{x})(j-\mu_{y})/\sigma_{x}\sigma_{y}\}^{3}\right]p(i,j)$ | A measure of the symmetry distribution of the gray levels |

*SGLDM is based on the estimation of the second order conditional probability density P (*i*, *j*, *d*, *Ө*). The value of P at location (*i*, *j*, *d*, *Ө*) of the SGLD matrix signifies the probability that two different resolution cells, in a specified orientation *Ө* from the horizontal and specified distance *d* from each other, have gray level values *i* and *j*, respectively.

* In all equations, $p(i,j)$is the $(i,j)^{th}$ entry of the normalized SGLDM, N is the total number of gray levels in the image; and µ_x_, µ_y_, and σ_x_, σ_y_ represent the mean and standard deviation of the row and column sums of the SGLDM, respectively.

**The gray level sum distribution is given by $p_{x+y}(k)=\sum_{\begin{matrix} i=1 \\ i+j=k \end{matrix}}^{N} \sum_{j=1}^{N} p(i,j), k=2,3...,2N$; it is related to the distribution of sum of the co-occurring pixels in the image.

*** The gray level difference distribution is given by $p_{x-y}(k)=\sum_{\begin{matrix} i=1 \\ \left|i-j\right|=k \end{matrix}}^{N} \sum_{j=1}^{N} p(i,j), k=0,1,2,...,N-1;$it is related to the distribution of difference between co-occurring pixels in the image.

HXY is the entropy and *HX* and *HY* are the entropies of *p_x_* and *p_y_*.
